# Clinical and Epidemiological Profiles of Patients with End Stage Kidney Disease on Dialysis at Dialysis Centers in Addis Ababa, Ethiopia

**DOI:** 10.4314/ejhs.v33i3.13

**Published:** 2023-05

**Authors:** Lissane Seifu, Seifemichael Getachew, Bezaye Abebe, Zerihun Debay

**Affiliations:** 1 Department of Internal Medicine, College of Health Science, Addis Ababa University, Addis Ababa, Ethiopia; 2 Department of Internal Medicine, St. Paul's Hospital Millennium Medical College, Addis Ababa, Ethiopia; 3 Department of Pediatrics, College of Health Science, Addis Ababa University, Addis Ababa, Ethiopia; 4 Santé Medical Center, Private Clinic, Addis Ababa, Ethiopia

**Keywords:** End stage kidney disease, Epidemiology, Dialysis, Ethiopia

## Abstract

**Background:**

End-stage kidney disease is increasing worldwide, primarily in the developing countries. It is affecting mainly the productive age group. Though the prevalence of the disease is increasing in Ethiopia, there are few studies. We therefore sought to describe the clinical and epidemiological characteristics of hemodialysis patients.

**Methods:**

A descriptive cross-sectional study was conducted at 17 hemodialysis centers in Addis Ababa. Patients who received hemodialysis for at least three months were included in the study. Socio-demographic and clinical data were collected via questionnaires from October to November 2021.

**Results:**

This study involved 318 participants with males making up 197 (61.9%) of the participants. Among the total, 248 (78.0%) were in the age group of 20 to 60 years and 155 (48.7%) were unemployed. Hypertension and diabetes mellitus were the major risk factors for end-stage kidney disease. The mean duration of dialysis was 2.26 years. The frequency of dialysis was twice weekly in 150 (47.2%) of the patients and thrice weekly in 138 (43.4%) of the patients. Arterio veneous fistula was used in approximately three-quarters of patients, i.e. 237 (74.5%). The majority of the patients on hemodialysis, 193 (60.7%), pay their own expenses, and 52 (16.4%) of the patients received hemodialysis at public hemodialysis centers. More than two-third of the patients were not in the process of undergoing a kidney transplant due to a variety of factors, including inability to find a donor, 106 (47.7%), being unfit for surgery, 56 (25.2%), and financial constraints, 38 (17.1%).

**Conclusion:**

The majority of the patients on dialysis were men, middle-aged, and unemployed. The majority of the patients underwent dialysis in private institutions and were self-funded, and most of them had inadequate dialysis doses. The inability to find a living donor was the most difficult aspect of undergoing kidney transplantation.

## Introduction

End-stage kidney disease (ESKD) is a steadily increasing global health and healthcare burden, primarily in developing countries, such as Africa, and mainly affects productive age groups. Understanding the epidemiology of ESKD patients is an important step in the prevention and management process (1, 3). In Africa, data on the burden of chronic kidney disease (CKD) are gradually becoming available, but ESKD patient profiles are not yet fully defined in most African countries, including Ethiopia (2, 4).

ESKD is associated with both non-communicable diseases (like diabetes mellitus and hypertension) and communicable diseases (such as infectious glomerulopathies and human immunodeficiency virus) (2, 5–10), and the etiology varies by country. Diabetes and hypertension account for greater than 70% of patients with CKD receiving hemodialysis in Europe and less than 50% in the United States.

In sub-Saharan Africa, CKD and ESKD primarily affect young and middle-aged people (20–50 years old), with a high proportion of males, whereas the opposite is true for older populations in developed countries (2, 5). Data on the epidemiology and dialysis treatment of ESKD in Ethiopia are sparse, and knowledge of the extent of kidney disease is very limited. Studies conducted on diabetes and hypertension patients in Northwest Ethiopia and Tigray teaching hospitals found that 17.3% of diabetic patients and 22.2% of hypertensive patients had CKD based on an estimated glomerular filtration rate (eGFR) of 60 mL/min/1.73 m2, respectively (11, 12). The care for ESKD patients in developing countries is challenging due to the large numbers of patients and inadequate facilities (4, 5). Renal replacement therapies (RRT) are essential for the survival of ESKD patients. Although kidney transplantation is the ideal treatment for patients with ESKD, maintenance hemodialysis (MHD) is the most common and widely used method worldwide. The demand for MHD is high in both developed and developing countries, but there are differences in the accessibility of modalities in developing versus developed countries (3–5).

In Ethiopia, kidney transplantation is practiced only in one government hospital, and the service is limited to a few patients. Thus, the majority of patients with ESKD remain dialysis-dependent. Maintenance hemodialysis is the most commonly available modality in Ethiopia, with services expanding over the last two decades, but mainly in the private sector. The cost of MHD is so high that it appears to be similar with other developing and developed countries (3–5). In Addis Ababa, the cost per session is Ethiopian Birr (ETB) 1500-3000. Therefore, in this study, we described he socio-demographic and epidemiology of ESKD patients on hemodialysis.

## Materials and Methods

**Study period, area, and design**: There are 20 dialysis centers (4 public and 16 private) in Addis Ababa. Of these, one public dialysis center is giving service to only patients with Acute Kidney Injury (AKI). A cross-sectional descriptive study was performed in 17 dialysis centers (3 public and 14 private) in Addis Ababa from October 21 to November 20, 2021.

**Study Population**: Patients with ESKD who were on dialysis in healthcare facilities during the study period constituted the study population.

**Sample Size and Sampling Technique**: Non-probability convenience sampling method was used to enroll every consecutive patient during their outpatient dialysis sessions and patients hospitalized for a complication in selected centers during the study period. Data was collected for two consecutive days at each dialysis center. The two private centers that refused to participate in the study and a public center that provided dialysis to only patients with AKI were excluded. Patients who were critically ill, unable to respond, or diagnosed within the previous three months were also excluded.

**Data collection tools and procedures**: Data were collected by trained renal nurses by interviewing patients and reviewing their medical records using structured questionnaires developed in Amharic and English versions. The socio-demographic characteristics, medical and clinical profiles were included in the questionnaires.

**Operational definitions**: All comorbidities were defined as present if they were documented in the medical records by the treating physician. All patients on hemodialysis for more than three months were considered to have ESKD.

**Data quality assurance, processing, and management**: The collected data were validated and exported to SPSS 26 for analysis. Descriptive statistics included mean with SD for continuous variables, and frequency and percentage were used for categorical data. Texts and tables were used to present the results.

**Ethical considerations**: Ethical approval was obtained from the Santé Medical College Institutional Review Board, and permits were obtained from each medical facility. Informed consent was obtained from each study participant, and the data were analyzed anonymously.

## Results

A total of 619 patients received dialysis in the 17 public (98 participants) and private (521 participants) facilities. However, 301 individuals were excluded from the study because they were either not available during the data collecting period, or were critically ill, or had only recently been diagnosed (<3 months). Among the 318 study participants recruited for the study, 197 (61.9%) were men. The age group between 31 and 40 years is the most numerous, accounting for 77 (24.2%), followed by ages 41 to 50 years, 65 (20.4%), with minimum and maximum ages of 14 and 79 years, respectively. More than half of the study participants were married. Almost half of the patients were high school level, 91 (28.6%) and degree holders, 72 (22.6%). Around one-fourth (27.4%) of the participants were employed. Most of the participants, 284 (89.3%), were from Addis Ababa City (Table 1).

**Table 1:** Socio-demographic characteristics of end stage kidney disease patients on hemodialysis at dialysis centers in Addis Ababa, Ethiopia from October 21 to November 20, 2021

Variables	Category	Number	Percent
**Gender**	Male	197	61.9
	Female	121	38.1
**Age**	<20	11	3.5
	20-30	51	16.0
	31-40	77	24.2
	41-50	65	20.4
	51-60	55	17.3
	61-70	39	12.3
	>70	20	6.3
**Educational Status**	Cannot read and write	20	6.3
	Read and write only	30	9.4
	Elementary school	30	9.4
	High school	91	28.6
	Diploma	68	21.4
	Degree	72	22.6
	Postgraduate	7	2.2
**Current Occupation**	Student	17	5.3
	Government employee	35	11.0
	Non-governmental organization employee	6	1.9
	Self employed	46	14.5
	Unemployed	155	48.7
	Retired	40	12.6
	Others	19	6.0
**Current Living Address**	Addis Ababa	284	89.3
	Out of Addis Ababa	34	10.7

Hypertension was found to be the most common risk factor of ESKD, 105 (33.0%), followed by concomitant hypertension and diabetes mellitus, 67 (21.1%), and diabetes mellitus alone 38 (11.9%); whereas, 79 (24.8%) of the participants had no clearly defined risk factors. Cardiac diseases were the most common comorbidities, 25 (7.9%), followed by tuberculosis (TB) and post-TB complications, 16 (5.0%), and human immunodeficiency virus (HIV), 9 (2.8%) (Table 2).

**Table 2:** Risk factors and comorbidities of end stage kidney disease patients on hemodialysis at dialysis centers in Addis Ababa, Ethiopia from October 21 to November 20, 2021

Variables	Category	Number	Percent
**Risk factors**	Hypertension	105	33.0
	Diabetes Mellitus	38	12.0
	Hypertension and Diabetes Mellitus	67	21.1
	Obstructive uropathy	13	4.1
	Human Immunodeficiency Virus	9	2.8
	Chronic glomerulonephritis	7	2.2
	Unknown factors	79	24.8
**Comorbidities**	No	249	78.3
	Cardiac diseases	25	7.9
	Tuberculosis/post tuberculosis complication	16	5.0
	Human Immunodeficiency Virus	9	2.8
	Chronic liver disease	4	1.3
	Malignancy	3	0.9
	Bronchial asthma	1	0.3
	Others	11	3.5

The mean duration of dialysis was 2.26 years, and the majority of the patients, 211 (66.4%), were on dialysis for more than 12 months. Only 142 (44.6%) of the patients received dialysis three or more times weekly. The most commonly used vascular access was arteriovenous fistula (AVF), 237 (74.5%), followed by permanent catheterization, 34 (10.7%) (Table 3).

**Table 3:** The duration and weekly frequency of hemodialysis for end stage kidney disease patients at dialysis centers in Addis Ababa, Ethiopia from October to November 20, 2021

Variables	Category	Number	Percent
**Duration on hemodialysis**	<1 year	107	33.6
	1-2 years	66	20.8
	3-5 years	106	33.3
	6-10 years	34	10.7
	>10years	5	1.6
**Frequency of hemodialysis sessions**	≤1x/week	26	8.2
	2x/week	150	47.1
	3x/week	138	43.4
	>3x/week	4	1.3
**Current vascular access**	Arterio-veneous fistula	237	74.5
	Permanent catheter	34	10.7
	Temporary venous catheter	28	8.8
	Arterio-veneous graft	19	6.0

Most of the patients, 266 (83.6%), had hemodialysis at private dialysis centers. The majority of patients on hemodialysis, 193 (60.7%), paid their own expenses. More than two-thirds, 222 (69.8%), of patients were not in the process of undergoing kidney transplant due to varieties of factors, including inability to find a donor (47.7%), being unfit for surgery (25.2%), and financial constraints (17.1%). Nine patients (4.1%) had no knowledge of how and where to begin the kidney transplantation process (Table 4).

**Table 4:** Expense sponsorship and kidney transplantation process of patients with end stage kidney disease on hemodialysis at dialysis centers in Addis Ababa, Ethiopia from October 21 to November 20, 2021

Variables	Category	Number	Percent
**Patient distribution at hemodialysis facilities**	Government facility	52	16.4
	Private facility	266	83.6
**Expense sponsorship**	Self/family	193	60.7
	Government	45	14.2
	Civil Society organization/ Association	22	6.9
	Individual donor	22	6.9
	Non-governmental organization	9	2.8
	Others	27	8.5
**Kidney transplantation process**	Yes	96	30.2
	No	222	69.8
**The reason for not undergoing the kidney** **transplant procedure (n = 222)**	Could not find donor	106	47.7
	Unfit for surgery	56	25.2
	Financial reason	38	17.1
	No idea where to start the process	9	4.1
	Do not want to	5	2.3
	Others	8	3.6

Both at public and private dialysis centers, patients with a dialysis frequency below the minimum KDOQI recommended dose were comparable. AVG was slightly more practiced at public centers compared to the private, whereas AVF was more practiced at private dialysis centers (Table 5).

**Table 5:** A comparative exercise of patients with end stage kidney disease on hemodialysis at government and private dialysis centers in Addis Ababa, Ethiopia from October 21 to November 20, 2021

Variables	Category	Ownership of the Health Facility			
		
		Government		Private	
		n (52)	%	n (266)	%
Duration of hemodialysis	<1 Year	19	36.5	88	33.1
	1-2 Years	13	25.0	53	19.9
	2-5 Years	15	28.8	91	34.2
	5-10 Years	4	7.7	30	11.3
	>=10 years	1	1.9	4	1.5
Frequency of hemodialysis	< Once per week	0	0	7	2.6
sessions per week	Once per week	0	0	19	7.1
	Twice per week	28	53.8	122	45.9
	Three times per week	23	44.2	115	43.2
	> Three times per week	1	1.9	3	1.1
Current vascular access	Temporary veneous catheter	4	7.7	24	9.0
	Permanent catheter	6	11.5	28	10.5
	Arterio-veneous fistula	36	69.2	201	75.6
	Arterio-veneous graft	6	11.5	13	4.9
Kidney transplantation	Yes	16	30.8	82	30.8
process	No	36	69.2	184	69.2

## Discussion

According to this study, hypertension and diabetes mellitus were the two main risk factors for ESKD. The majority of patients received lesser doses of dialysis than what KDOQI recommends as the minimal dose, and most of the patients underwent hemodialysis at private dialysis facilities. A high proportion of patients were not being considered for kidney transplantation. In Ethiopia, kidney transplantation is practiced only in one governmental hospital, which serves a limited number of patients and needs a living donor. Hence, the inability to find a donor was a major challenge for those who underwent kidney transplantation, and finding funding to travel abroad for transplantation was another critical issue.

The majority of hemodialysis patients were men, with the male-to-female ratio of 1.6:1, which is similar to other studies conducted in Nepal, Oman, China, Burkina Faso, and Sudan (8, 13–17). A facility-based cross-sectional study conducted in Addis Ababa City and Amhara Region of Ethiopia in 2018 also showed a predominance of men (18). Almost two-thirds (64.1%) of patients were under 50 years old, and the mean age was 44.7 (14.9) years. This is similar to the results of several studies conducted at tertiary hospitals in Asian and African countries (9, 13–15, 18, 19).

In this study, hypertension was found to be the most common risk factor for ESKD, followed by diabetes mellitus, which is consistent with the findings of many earlier studies (6, 8, 9, 14). Most systematic reviews and hospital-based cross-sectional studies undertaken in Africa also found that hypertension was the major cause of ESKD, followed by chronic glomerulonephritis (CGN) (4, 5, 13, 15, 16, 19).

The vast majority of, 279 (87.7%), patients had been on dialysis for five years or less, which was slightly higher than the 2006 cross-sectional study in Aleppo (9) but comparable to the study in Burkina Faso (15). The majority of patients in our study (55.4%) received less than thrice weekly dialysis, although according to KDOQI guidelines, the recommended minimum dialysis dose is three times a week (20). This is similar to other studies that showed inadequate dialysis in many patients (3, 5). The reasons for this may be insufficient facilities, high unemployment rate, and limited number of free or government-funded dialysis centers. This insufficient dialysis dose can further lead to a decrease in the quality of life of such patients.

Arterio-veneous fistula (AVF) was the preferred mode of vascular access, and around three-fourth of the patients used AVF followed by permanent catheters (10.7%) and temporary catheters (8.8%). This finding is consistent with the results of a 2010 systematic review in ten countries, which found that, on average, 71.4% of hemodialysis patients used an AVF, followed by an indwelling catheter (10.1%) and a temporary catheter (8.1%) (7). Other studies also concluded that AVF was the most commonly used and preferred mode of vascular access for hemodialysis (3, 9, 18).

In this study, only 16.4% of patients underwent hemodialysis at public hemodialysis facilities, in contrast to a study conducted in Aleppo, where 70.4% of patients received dialysis at public centers (9). This is because of the inadequate number of public dialysis facilities in Ethiopia. This study showed that the majority of patients on hemodialysis paid their own expenses. Meanwhile, according to a study conducted in Kazakhstan, all the costs of RRT were fully covered by the country (10). These differences may be due to the financial difficulties faced by most African countries.

The inability to find a donor (47.7%) was discovered to be the most difficult challenge in patients who were not undergoing the kidney transplantation process. This is because, in Ethiopia, kidney transplantation is practiced only in one public hospital, the service is limited to a few patients, and the patients need living donors. Studies have shown that transplant rates in sub-Saharan countries are low, with the majority of transplants coming from living donors (4, 5). However, over the 25-year period under consideration, South Africa performed 7191 kidney transplants, with 58.3% of the donated kidneys coming from deceased donors (21). According to a study conducted in Bangladesh, the main barriers to organ donation were the opposition of Muslim Orthodox clerics', as well as other cultural and legal difficulties (22). A research conducted at Jimma University Medical Center, after discovering that the majority of medical professionals were well aware of cadaveric organ donation (COD), but that their attitude and willingness to body donation were significantly lower than their knowledge, recommended intensive education to raise the attitude and willingness in order to develop COD programs in Ethiopia (23).

The limitation of the current study is that it only included patients who had dialysis in Addis Ababa, not those who received hemodialysis in the country's regional states. The majority of patients on hemodialysis were males, married, working age groups, and unemployed. Hypertension and diabetes continue to be the major risk factors for ESKD in patients on hemodialysis. AVF was the preferred mode of vascular access for hemodialysis. The majority of patients underwent dialysis in private institutions and were self-funded. Most of the patients with ESKD had inadequate dialysis doses. The impossibility of finding a donor and budgetary constraints were the main reasons for rejecting kidney transplant. Therefore, future studies should analyze Ethiopia's religious, cultural, and legal barriers to organ donation and the justifications against using cadaveric organ transplants in Ethiopia.
